# Lutein Inhibits the Migration of Retinal Pigment Epithelial Cells via Cytosolic and Mitochondrial Akt Pathways (Lutein Inhibits RPE Cells Migration)

**DOI:** 10.3390/ijms150813755

**Published:** 2014-08-08

**Authors:** Ching-Chieh Su, Chi-Ming Chan, Han-Min Chen, Chia-Chun Wu, Chien-Yu Hsiao, Pei-Lan Lee, Victor Chia-Hsiang Lin, Chi-Feng Hung

**Affiliations:** 1 Graduate Institute of Applied Science and Engineering, Fu Jen Catholic University, New Taipei City 24205, Taiwan; E-Mail: suk.ccsu@gmail.com; 2 Department of Internal Medicine, Cardinal Tien Hospital, New Taipei City 23148, Taiwan; 3 School of Medicine, Fu Jen Catholic University, New Taipei City 24205, Taiwan; E-Mail: m212092001@tmu.edu.tw; 4 Department of Ophthalmology, Cardinal Tien Hospital, New Taipei City 23148, Taiwan; 5 Graduate Institute of Applied Science and Engineering, Fu Jen Catholic University, New Taipei City 24205, Taiwan; E-Mail: 056489@mail.fju.edu.tw; 6 Department of Life Sciences, Fu Jen Catholic University, New Taipei City 24205, Taiwan; E-Mail: bobwu80113@gmail.com; 7 Department of Nutrition and Health Science, Chang Guang University of Science and Technology, Taoyuan 33303, Taiwan; E-Mail: mozart@gw.cgust.edu.tw; 8 Research Center for Industry of Human Ecology, Chang Gung University of Science and Technology, Taoyuan 33303, Taiwan; 9 Slone Epidemiology Center, Boston University, Boston, Massachusetts, United States of America, Boston, MA 02215, USA; E-Mail: peilan@bu.edu; 10 Department of Urology, E-Da Hospital, Taiwan and School of Medicine for International Students, I-Shou University, Kaohsiung 84001, Taiwan; E-Mail: ed102161@edah.org.tw

**Keywords:** Akt translocation, mitochondria, lutein, retinal pigment epithelium

## Abstract

During the course of proliferative vitreoretinopathy (PVR), the retinal pigment epithelium (RPE) cells will de-differentiate, proliferate, and migrate onto the surfaces of the sensory retina. Several studies have shown that platelet-derived growth factor (PDGF) can induce migration of RPE cells via an Akt-related pathway. In this study, the effect of lutein on PDGF-BB-induced RPE cells migration was examined using transwell migration assays and Western blot analyses. We found that both phosphorylation of Akt and mitochondrial translocation of Akt in RPE cells induced by PDGF-BB stimulation were suppressed by lutein. Furthermore, the increased migration observed in RPE cells with overexpressed mitochondrial Akt could also be suppressed by lutein. Our results demonstrate that lutein can inhibit PDGF-BB induced RPE cells migration through the inhibition of both cytoplasmic and mitochondrial Akt activation.

## 1. Introduction

Proliferative vitreoretinopathy (PVR) and proliferative diabetic retinopathy (PDR) are two main causes of blindness in developed countries [1], and the hallmark of PVR is the growth and contraction of cellular membranes within the vitreous cavity and on both surfaces of the retina. Cells of the retinal pigment epithelium (RPE), a monolayer of pigmented cells located between the neurosensory retina and the choroid [2], de-differentiate, proliferate, and migrate onto the surfaces of the sensory retina during the course of PVR, which may lead to the formation of contractile epiretinal and vitreous membranes, and further lead to recurrent retinal detachment [3].

Various growth factors and cytokines have been implicated in the pathogenic mechanisms of PVR. Among them, platelet-derived growth factor (PDGF) has been suggested to play an important role in the induction of events that contribute to PVR [4,5]. While PDGF ligands and their receptors are ubiquitous in proliferative retinal membranes [6], phosphoinositide 3-kinase (PI3K) and Akt (protein kinase B) were the main signaling enzymes required for PDGF receptors to mediate PVR [7,8]. PDGF can also stimulate the expression of vascular endothelial growth factor (VEGF) from RPE after activating the extracellular signal-regulated kinases 1/2 (ERK1/2; p44/p42 MAPK), p38 and Akt proteins [9]. Both PDGF and VEGF play a part in the progression of epiretinal membranes [10]. Akt is a critical signaling node in the cytosolic compartment within cells. Its role in the nuclear compartment has also been investigated [11]. In addition, its ability to translocate into mitochondria after insulin stimulation in cardiomyocytes was also reported recently [11].

Carotenoids, synthesized only in plants, can be split into two classes, xanthophylls and carotenes. While β-carotene and lycopene belongs to carotenes, lutein and zeaxanthin are two major forms of xanthophylls [12,13]. Studies have shown that β-carotene can be converted to vitamin A in RPE, and inhibits the migration and proliferation of RPE cells [14,15]. In our previous study, we found that lycopene can inhibit PDGF-induced RPE cell migration [16]. Lutein possesses biological antioxidant activity, and it is a major component of carotenoids with non-provitamin A activity [13]. Its capability of inhibiting increased PI3K activity and Akt phosphorylation after oxidative stress in macrophages has been reported [17]. However, the inhibitory effect of lutein on the migration of RPE is still not known.

In this study, we investigated the effect of lutein on PDGF-induced RPE cell migration and its relative mechanisms. We also hypothesized that the phospho-Akt after PDGF stimulation will translocate into mitochondria in RPE cells. Finally, we conducted some studies regarding the effect of mitochondrial Akt on RPE migration.

## 2. Results and Discussion

### 2.1. Lutein Can Inhibit PDGF-Induced Migration of RPE Cells via an Akt Pathway

**Figure 1:**
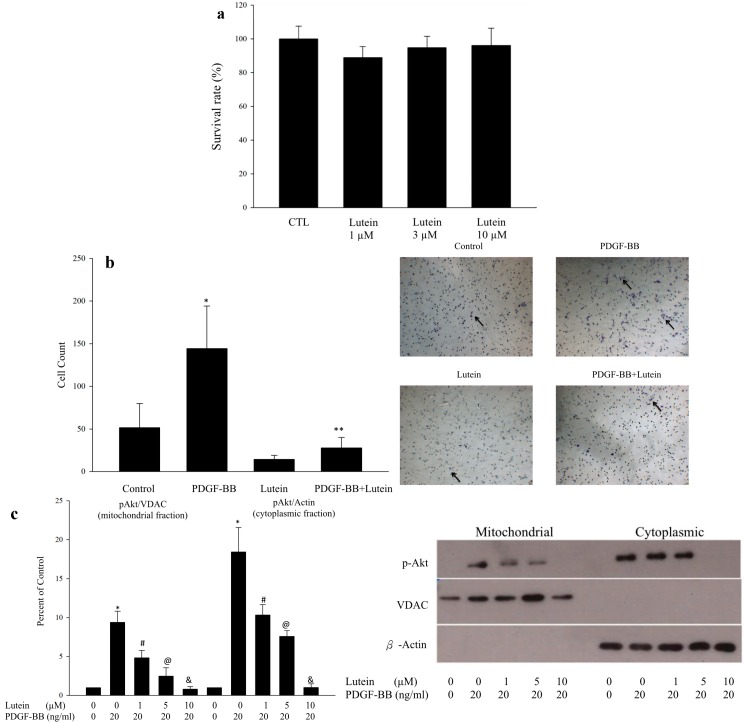
(**a**) MTT assay of lutein. No significant cytotoxicity of lutein was noted up to concentrations of 10 μM; (**b**) Transwell migration assay and cell count bar graph. PDGF can increase migration of RPE cells, and lutein at the concentration of 10 μM can inhibit the PDGF-induced migration of RPE cells. Transwell migration assay was performed as described in the Materials and Methods section. Migration was quantified by counting the number of stained cells per 100 high power field (HPF) in images taken with a phase-contrast microscope. Black arrow indicates the migrated RPE cells. Cell count bar graph was made from four independent repetitions of the experiments. * *p* < 0.001 *vs.* control, ** *p* < 0.001 *vs.* PDGF-BB; (**c**) The Western blot showed that phospho-Akt increases in both mitochondrial and cytoplasmic compartment under PDGF-BB stimulation (20 ng/mL). The stimulatory effect was inhibited by lutein in a dose dependent manner. The bar graph represented the data summarized from four experiments. The data were normalized to the contents of voltage-dependent anion channel(VDAC) in mitochondrial fraction and actin in cytoplasmic fraction. * *p* < 0.01 *vs.* control; # *p* < 0.01 *vs.* lutein 0μM; @ *p* < 0.05 *vs.* lutein 1μM; & *p* < 0.05 *vs.* lutein 5μM.

As lycopene can inhibit the PDGF-induced RPE cell migration through the suppression of PI3K/Akt and MAPK pathways [16], we investigated the effects of lutein, another carotenoid, on the RPE cells migration induced by PDGF. In the beginning, the MTT (3-(4,5-dimethylthiazol-2-yl)-2,5-diphenyltetrazolium bromide) assay was performed to determine the toxicity of lutein. Different concentrations of lutein were chosen and no significant toxicity was noted up to concentrations of 10 μM (Figure 1a). Then the transwell migration assay was used to determine the effects of lutein on the migration of RPE cells. We found that PDGF stimulation increased the migration of RPE cells, and this increase was reduced by lutein (10 μM) (Figure 1b).

**Figure 2:**
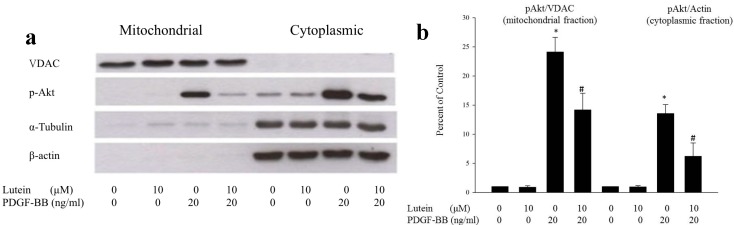
Effects of PDGF and lutein on RPE cells (**a**) The Western blot showed the stimulatory effect of PDGF at the concentration of 20ng/mL, and the inhibitory effect of lutein at the concentration of 10μM on phopho-Akt in both the cytoplasmic and mitochondrial compartments. In this diagram, α-tubulin and β-actin represent the markers of cytosolic fraction of RPE cells, and voltage-dependent anion channel (VDAC) represents the marker of mitochondria fraction. The results presented are representative of five independent experiments; (**b**) The bar graph represented the data summarized from five experiments. These data were normalized to the contents of VDAC in mitochondrial fraction and actin in cytoplasmic fraction. * *p* < 0.01 *vs.* control; # *p* < 0.01 *vs.* lutein 0 μM.

Using Western blot, we also found that the inhibitory effect of lutein on Akt phosphorylation within both cytoplasmic compartments and mitochondrial compartments was dose-dependent (Figure 1c). To validate our observation, several experiments were further carried out. We confirmed that PDGF can increase phospho-Akt both in the cytoplasmic and mitochondrial fractions, which is in agreement with results found in cardiomyocytes after insulin stimulation [18]. Additionally, the phosphorylation of Akt was reduced by lutein in both the mitochondrial and cytoplasmic compartments. These results indicate that lutein can inhibit PDGF-induced translocation of phospho-Akt into mitochondria (Figure 2a,b), and that mitochondrial Akt activity may play an important role in RPE cell migration.

### 2.2. Ad-Mito-Akt Can Transduce RPE Successfully with Constitutively Activated Mitochondrial Akt

**Figure 3:**
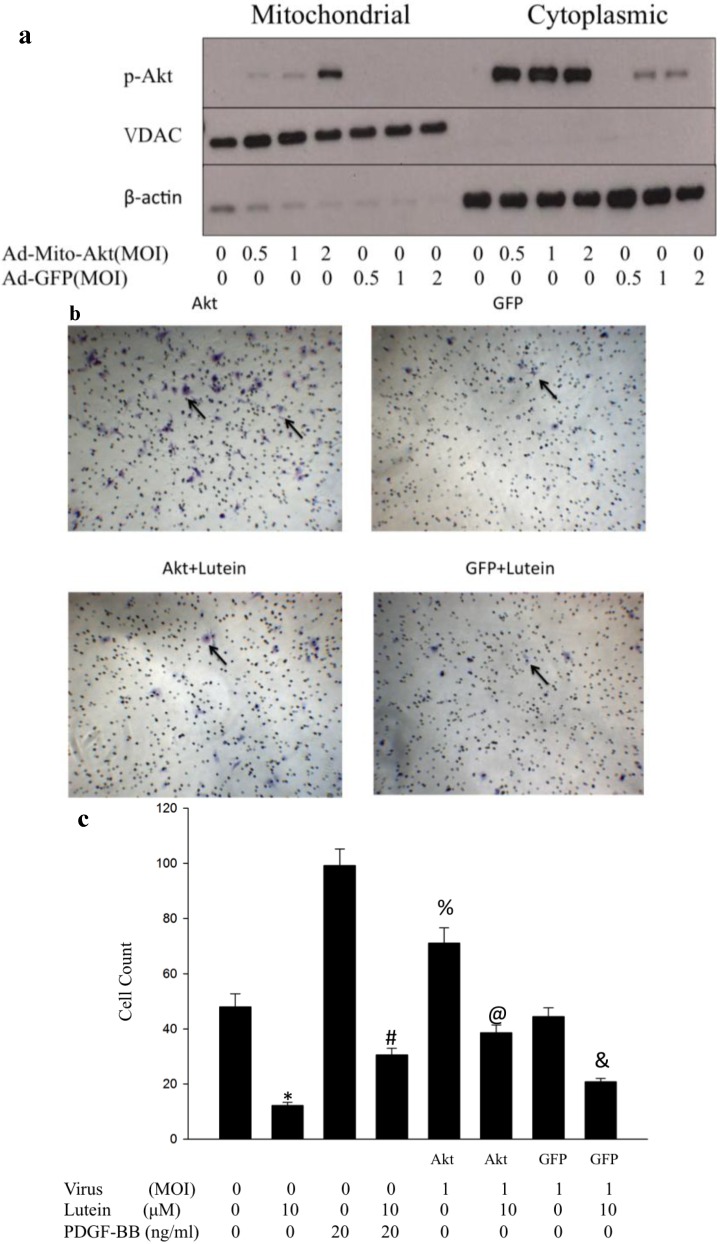
Effect of overexpressed mitochondrial Akt on RPE cell migration (**a**) The Western blot showed that Ad-Mito-Akt virus can overexpress mitochondrial phospho-Akt in RPE cells. Different multiplicity of infection (MOI), the ratio of virus to RPE cells, of Ad-Mito-Akt and Ad-GFP viruses were used to transduceRPE cells. As the MOI of Ad-Mito-Akt increased, phospho-Akt in the mitochondrial compartment increased. No phospho-Akt activity in the mitochondrial fraction was detected in Ad-GFP transduced RPE cells;(**b**) Transwell migration assay. The migration activity of Ad-Mito-Akt transduced RPE cells increased when compared with Ad-GFP transduced RPE cells. The increased migration activity of RPE cells transduced with Ad-Mito-Akt was inhibited by lutein in the concentration of 10 μM; (**c**) The bar graph showed the cell count of migrated RPE cells treated with or without PDGF, lutein, and with or without transduction using Ad-Mito-Akt or Ad-GFP. Data are expressed in mean ± SD of 5 independent experiments. * *p* < 0.001 *vs.* control; # *p* < 0.001 *vs.* PDGF 20 mg/dL and lutein 0 μM; @ *p* < 0.001 *vs.* Akt with MOI 1; & *p* < 0.001 *vs.* GFP with MOI 1; % *p* < 0.05 *vs.* control.

Ad-Mito-Akt was demonstrated to cause overexpression of mitochondrial Akt only [19]. In order to understand the function of phospho-Akt in mitochondria, we used Ad-Mito-Akt to increase the expression of activated Akt inside mitochondria. Western blots were performed using cultured RPE cells that were transduced with different concentration of viruses of Ad-Mito-Akt or Ad-GFP, our control group. We observed that the expression of mitochondrial phospho-Akt in RPE cells transduced with Ad-Mit-Akt increased as multiplicity of infection (MOI) increased. In contrast, no phospho-Akt could be detected in RPE cells transduced with Ad-GFP viruses (Figure 3a). These results demonstrate that Ad-Mito-Akt can successfully increase phospho-Akt inside mitochondria compared to Ad-GFP virus. We also observed a modest increase in cytoplasmic phospho-Akt when cells were transduced with Ad-GFP and a much larger increase when cells were transduced with Ad-Mito-Akt.

### 2.3. Overexpressed Mitochondrial Akt Can Increase Migration of RPE Cells. Lutein Can Successfully Inhibit Migration of Rpe Cells with or without Activation of Mitochondrial Akt

To determine the role of mitochondrial phospho-Akt on the migration of RPE cells, we used transwell migration assay and RPE cells trasduced with either Ad-Mito-Akt or Ad-GFP. Compared to the control group (RPE cells transduced with Ad-GFP), the migration activity of RPE cells transduced with Ad-Mito-Akt increased (Figure 3b,c), which indicates that the migration activity of RPE cells can be increased solely by increasing mitochondrial Akt. Furthermore, we found that the increased migration activity of RPE cells transduced with Ad-Mito-Akt was diminished by lutein.

The above experiments imply that mitochondrial Akt activity plays an important role in RPE cells migration. Lutein can decrease migration activity of RPE cells with or without overexpressed mitochondrial Akt activity.

### 2.4. Discussion

To the best of our knowledge, the role of mitochondrial Akt in RPE cell migration has not been elaborated. In this study, we demonstrated that the mechanism of the inhibitory effect of lutein on RPE cell migration was through the Akt pathway. Most importantly, we found that mitochondrial Akt plays an important role in RPE cell migration and it can be inhibited by lutein.

RPE cell migration is crucial in many kinds of eye diseases, such as age-related macular degeneration (AMD), proliferative vitreoretinopathy and proliferative diabetic retinopathy [10,20,21]. Various growth factors, like platelet-derived growth factor, vascular endothelial growth factors, insulin like growth factor (IGF) and epidermal growth factor (EGF), play a part in the pathogenesis [10]. Among them, platelet-derived growth factor (PDGF) has been suggested to play an important role in the induction of events that contribute to PDR [4,5]. Receptors of PDGF, VEGF, IGF and EGF belong to the family of receptor tyrosine kinase, a subclass of protein kinase. It can transfer the phosphate group attached to tyrosine on a protein to another protein in a cell. PI3K and Akt are the main downstream pathways of receptor kinases and are involved in the migration of RPE cell migration [9]. In previous studies we have shown that epigallocatechin gallate, resveratrol and lycopene can inhibit PDGF induced migration of RPE cells through suppression of the Akt pathway [16,22,23]. In this study, we demonstrate that lutein, another member of carotenoids, can also inhibit PDGF-induced migration of RPE cells via the Akt pathway. Furthermore, we found that mitochondrial Akt plays an important role in the migration of RPE cells and lutein has an inhibitory effect on it as well.

The serine/threonine kinase Akt, also named as protein kinase B (PKB), plays a vital role in cell signaling downstream of cytokines, growth factors, and other cellular stimuli. Akt is also involved in different cellular activities including cell survival, growth, proliferation, angiogenesis, metabolism and migration [24]. Previous studies have focused mostly on signaling downstream of Akt in the cytosolic compartment. Recently, activated Akt was found to be localized in the Golgi apparatus, nucleus and mitochondria [25], and the role of Akt in subcellular compartments has been better analyzed. Nuclear accumulation of Akt was reported to produce anti-apoptotic, anti-hypertrophic activities in cardiomyocytes [26,27]. In addition, it was first known in 2003 that Akt can translocate into mitochondria in response to adequate stimulation in SH-SY5Y human neuroblastoma cells and HEK 293 human embryonic kidney cells [28]. Studies showed that the location of mitochondrial Akt may not be identical in different cell types, and its function has not been fully understood. Akt was found mainly in the outer and inner mitochondrial membrane and to a lesser degree in the matrix [28] in SH-SY5Y human neuroblastoma cells, while it was located in intermembrane space, inner membrane and matrix in myocardial cells [29]. Glycogen synthase kinase-3β and β-subunit of ATP synthase were identified to be phosphorylated following stimulation of mitochondrial Akt [28]. Mitochondrial Akt was shown to have cardioprotective activities by preventing electrochemical gradient loss in mitochondria of cardiac muscle cells [18,30] and it was also shown to have anti-apoptotic ability in cardiomyocytes in response to hydrogen peroxide or doxorubicin [19]. In addition, mitochondrial Akt was believed to be related to energy production in cardiomyocytes. Insulin binding to insulin receptor, a kind of receptor tyrosine kinase, can restore myocardial complex V activity in the diabetic mice by stimulating translocation of Akt into mitochondria. The translocation effects of insulin can be blunt in the diet-induced diabetic myocardium, which implies the relationship of mitochondrial Akt and insulin resistance [11]. Activating PDGFα receptors, a family of receptor tyrosine kinase, by PDGF plays an important role in the development of experimental PVR [31,32]. In our experiment, PDGF was found to increase the phosphorylation of Akt in mitochondria, and the overexpressed mitochondrial Akt induced by Ad-Mito-Akt was associated with the migration of RPE cells. To our knowledge, its role in cell migration of RPE cells has not been reported in the literature; we suggest that its role in RPE cells, especially in the development of PVR, needs further investigation.

Lutein, along with zeathanthin, is the only xanthophyll, a kind of carotenoid, in the retina and lens [33]. Lutein can filter out photo-toxic short-wavelength visible light and minimize the effect of chromatic aberration [34]. It also has cytoprotective effects for RPE cells exposed to oxidative stress or UVB-mediated oxidative damage [35,36]. Studies have shown its capability in inhibiting PI3K activity and Akt phosphorylation in macrophages, vascular smooth muscle or colon cancer cells [17,37,38], while another study has revealed that carotenoids, including lutein, can enhance phosphorylation of Akt in human endothelial cells [39]. In our experiment, we found that lutein can suppress the migration of RPE cells by decreasing both cytoplasmic and mitochondrial Akt activity, with or without the activation of mitochondrial Akt. The possible mechanisms of mitochondrial Akt related to the migration, and how lutein works in mitochondrial Akt still need further clarification.

The limitations and assumptions in our experiments are associated with using Ad-Mito-Akt. Su *et al.* measured cytoplasmic activity of phospho-Akt by measuring phosphorylation of glycogen synthase 3-alpha (GSK-3α) when cardiomyocytes were transduced with Ad-Mito-Akt. In our experiments, we measured both the cytoplasmic and mitochondrial levels of phospho-Akt by Western blot. We assumed that the Akt regulation is similar in RPE cells and cardiomyocytes. The increased cytoplasmic level of phospho-Akt is possibly due to the overwhelming of the mitochondrial import machinery in RPE cells transduced with Ad-Mito-Akt or the activation of the PI3K/Akt pathway by virus infection in RPE cells transduced with Ad-GFP or Ad-Mito-Akt [40]. These data demonstrate that Akt activity requires more than the mere presence of Akt. Most mitochondria proteins are synthesized on cytosolic ribosomes except a few proteins that are synthesized within mitochondria. These pre-proteins with an amino-terminal are recognized by receptors of the outer membrane and translocated via the import pore of the outer membrane of mitochondria. The mitochondrial-targeting signal includes many positively charged, hydrophobic and hydroxylated amino-acid residuals. These presequences are likely to form an amphipathic α-helix with one positively charged surface and one hydrophobic surface [41]. Therefore, these pre-proteins in the cytosol could be bound by protein specific antibodies, but not be functional because of the different structure. In any event, the claim that mitochondrial Akt plays a role in RPE migration is dependent on the robustness of the observation of Su *et al.* Future work will be performed using constitutively active Akt or mitochondrial dominant negative Akt to further explore the role of this kinase.

Adhesion and migration of RPE cells are both important in the development of epiretinal membranes found in PVR. Inhibition of the Akt pathway may result in suppression of adhesion of RPE cells [42]. However, lycopene showed no effect on RPE cell adhesion in our previous study [16]. Therefore the role of cytoplasmic or mitochondrial Akt on adhesion of RPE cells, and the function of lutein on adhesion of RPE cells need further investigation.

## 3. Experimental Section

### Materials and Methods

Materials: Bovine serum albumin (BSA), aprotinin, leupeptin, phenylmethylsulfonyl fluoride (PMSF), sodium fluoride (NaF), and sodium orthovanadate were bought from Sigma Chemical Co. (St. Louis, MO, USA). Human plasma fibronectin was purchased from Invitrogen Life Technologies (Carlsbad, CA, USA). Antibody (Ab) against phospho-Akt was from Santa Cruz Biotechnology (Santa Cruz, CA, USA). Lutein was purchased from Extrasynthese (Genay, France). Human recombinant PDGF-BB was purchased from R&D systems (Minneapolis, MN, USA).

Cell cultures: Adult human retinal pigment epithelial cells (ARPE19), purchased from Food Industry Research and Development Institute (Hsinchu, Taiwan), were cultured in DMEM/F12 supplemented with 10% fetal calf serum (Gibco, Life Technologies, Carlsbad, CA, USA), 100 U/mL penicillin and 100 mg/mL streptomycin (Sigma Chemical Co., St. Louis, MO, USA) in a humidified incubator at 37 °C with 5% CO_2_. Cells reaching a 90%–95% of confluence were starved and synchronized in serum-free DMEM/F12 for 24 h before they were subjected to further analysis. Two recombinant adenoviruses, a control adenovirus that expresses green-fluorescent protein (Ad-GFP) and an adenoviral vector that expresses a mitochondria-targeting constitutively active mitochondrial-targeted Akt (Ad-Mito-Akt), were used in this study. The constitutively active Akt was created by mutating Thr308 and Ser473 to aspartic acid residues, which mimics phosphorylation and resulted in an active Akt construct and mitochondrial targeting was achieved by fusing a mitochondria targeting sequence (MSVLTPLLLRGLTGSARRLPVPRAKIHSL) to the N terminal [43]. Both viruses are gifts from Center for Diabetes Research and Treatment, University of California, Irvine.

Lutein treatment and PDGF incorporation: Lutein was dissolved in dimethyl sulfoxide (DMSO) to a concentration of 15 μM. This stock solution was prepared with minimal exposure to air and light, and stored at −80 °C. Immediately before the experiment, DMSO-lutein aliquots from the stock solution were added to the serum-free cell culture medium to a final concentration of 1, 5, 10 μM. For the Transwell migration assays and the Western blot analysis, the serum-free cell culture medium with various concentrations of lutein were all pre-incubated with or without PDGF-BB (20 ng/mL) at 37 °C for 30 min.

Preparation of mitochondria and cytosolic proteins: To prepare the mitochondrial fractions, cells were harvested in mitochondria isolation buffer (20 mM HEPES-KOH at pH 7.2, 10 mM KCl, 1.5 mM MgCl_2_, 1.0 mM sodium EDTA, 1.0 mM sodium EGTA, 1.0 mM dithiothreitol, and 250 mM sucrose), supplemented with protease/phosphatase inhibitors (3 μg/mL aprotinin, 3 μg/mL leupeptin, 2 mM phenylmethylsulfonyl fluoride, 20 mM NaF, 10 mM sodium pyrophosphate (NaPP), and 2 mM Na_3_VO_4_). After incubated on ice for 30 min, the cells were homogenized with 20 strokes of loose pestle and 50 strokes of tight pestle with a homogenizer. The nuclei and cell debris were removed by centrifugation at 1000× *g* for 15 min at 4 °C and the supernatants were collected for centrifugation at 10,000× *g* for another 15 min at 4 °C. The mitochondrial fractions obtained were resuspended with mitochondria isolation buffer, and the supernatants containing the cytosolic fractions were further centrifuged at 100,000× *g* for 1 h at 4 °C to collect cytosolic proteins. Both cytosolic and mitochondrial fractions were stored at −80 °C until further analysis. The protein concentrations were determined by the Bradford method.

Transwell migration assay: Transwell migration assay with ARPE19 cells were performed by using a modified Boyden chamber model (Transwell apparatus, 8.0 mm pore size, Costar). For detection of RPE cell migration in the Transwell, the lower face of each polycarbonate filter (Transwell insert) was coated with fibronectin (0.3 mg) for 30 min in the laminar flow hood, and the lower chambers were filled with 0.6 mL of serum-free medium or PDGF-BB (20 ng/mL)-containing medium pre-incubated with various concentrations of lutein. RPE cells (5 × 10^4^ cells, 200 μL) were plated to the upper chamber. After 5 h of incubation, all non-migrant cells were removed from the upper faces of the Transwell membranes with a cotton swab and migrated cells were fixed and stained with 0.5% toluidene blue in 4% PAF. Migration was quantified by counting the number of stained cells per 100 high power field (HPF) in images taken with a phase-contrast microscope (Leica DMIL1).

Western blot analysis of Akt: ARPE19 cells cultured on 6 cm dishes were starved for 24 h and then treated with various concentrations of lutein which were pre-incubated with or without PDGF-BB (20 ng/mL) at 37 °C for 30 min. They were then lysed in a mitochondria isolation buffer. After isolation of mitochondria, protein contents were quantified in both cytosolic and mitochondrial compartments by a Pierce protein assay kit (Pierce, Rockford, IL, USA). Total protein was separated by electrophoresis on 8% SDS-polyacrylamide gels. The proteins were then electroblotted onto PVDF membranes and probed using the specific antibodies mentioned. Immunoblots were detected by enhanced chemiluminescence.

## 4. Conclusions

In summary, this study demonstrates the mitochondrial translocation of Akt in response to PDGF stimulation. Activated mitochondrial Akt is associated with cell migration of RPE cells. Lutein can diminish the migration effect of activated mitochondrial Akt. Further exploration of the function of Akt within mitochondria should provide more information about its role inside mitochondria.
